# Supplementary material for: Climate zones are a key component of the heterogeneous presentation of malaria and should be added as a malariometric for the planning of malaria elimination
Source: PLOS Glob Public Health. 2023 Jun 28;3(6):e0001878. doi: 10.1371/journal.pgph.0001878 (PMC10306175; doi:10.1371/journal.pgph.0001878)
Supplement: S1 Text — Table A: Description and temperature/precipitation characteristics of the different Koppen-Geiger Climate classes and sub-classes. Table B: Malaria burden comparison between subtypes of temperate region. Table C: Malaria burden comparison between subtypes of tropical regions. (DOCX) [file pgph.0001878.s001.docx]

**S1 Text**

**Table A: Description and temperature/precipitation characteristics of the different Koppen-Geiger Climate classes and sub-classes**

| Climate Type | Sub-Category | Description | Temperature/Precipitation Characteristics |
| --- | --- | --- | --- |
| Tropical Climate (A) | Af | Tropical Rainforest | Rainforest |
|  | Am | Tropical Monsoon | Monsoon |
|  | As | Tropical Dry Savannah | Dry, Savannah |
|  | Aw | Tropical Wet Savannah | Wet, Savannah |
| Arid Climate (B) | BWh | Hot Desert | Arid desert, hot |
|  | BWk | Cold desert | Arid desert, cold |
|  | BSh | Hot Semi-Arid Steppe | Steppe, hot |
|  | BSk | Cold Semi-Arid Steppe | Steppe, cold |
| Temperate Climate (C) | Csa | Hot summer Mediterranean | Temperate, dry-hot summer |
|  | Csb | Warm summer Mediterranean | Temperate, dry-warm summer |
|  | Csc | Cold summer Mediterranean | Temperate, dry-cold summer |
|  | Cwa | Monsoon humid subtropical | Temperate, dry winter, hot summer |
|  | Cwb | Subtropical highland | Temperate, dry winter, warm summer |
|  | Cwc | Cold Subtropical Highland | Temperate, dry winter, cold summer |
|  | Cfa | Humid subtropical | Temperate, humid, hot summer |
|  | Cfb | Temperate Oceanic | Temperate, humid, warm summer |
|  | Cfc | Subpolar Oceanic | Temperate, humid, cold summer |
| Cold Climate (D) | Dsa | Mediterranean-influenced hot-summer humid continental | Cold, dry-hot summer |
|  | Dsb | Mediterranean-influenced warm-summer humid continental | Cold, dry-warm summer |
|  | Dsc | Mediterranean-influenced subarctic climate | Cold, dry-cold summer |
|  | Dsd | Mediterranean-influenced extremely cold subarctic climate | Cold, dry summer, very cold winter |
|  | Dwa | Monsoon-influenced hot-summer humid continental | Cold, dry winter, hot summer |
|  | Dwb | Monsoon-influenced warm-summer humid continental | Cold, dry winter, warm summer |
|  | Dwc | Monsoon-influenced subarctic | Cold, dry winter, cold summer |
|  | Dwd | Monsoon-influences extremely cold subarctic | Cold dry winter, very cold winter |
|  | Dfa | Hot-summer Humid continental | Cold humid, hot summer |
|  | Dfb | Warm-summer Humid continental | Cold humid, warm summer |
|  | Dfc | Subarctic | Cold humid, cold summer |
|  | Dfd | Extremely cold Subarctic | Cold humid, very cold winter |
| Polar Climate (E) | ET | Tundra | Polar, tundra |
|  | EF | Icecap | Polar Forest |

**Table B: Malaria burden comparison between subtypes of temperate regions**

| **Year** | **Sub Types** | **Temperate Districts with** | | **p-value** |
| --- | --- | --- | --- | --- |
|  |  | **API<1** | **API>=1** |  |
| 2015 | Cfa/Csa/Cwb  Cwa | 9(81.82%)  191(73.75%) | 2(18.18%)  68(26.25%) | 0.424 |
| 2016 | Cfa/Csa/Cwb  Cwa | 11(100%)  201(77.61%) | 0(0.00%)  58(22.39%) | 0.128 |
| 2017 | Cfa/Csa/Cwb  Cwa | 11(100%)  217(83.78%) | 0(0.00%)  42(16.22%) | 0.223 |
| 2018 | Cfa/Csa/Cwb  Cwa | 11(100%)  232(89.58%) | 0(0.00%)  27(10.42%) | 0.609 |
| 2019 | Cfa/Csa/Cwb  Cwa | 11(100%)  245(94.59%) | 0(0.00%)  14(5.41%) | 0.550 |
| 2020 | Cfa/Csa/Cwb  Cwa | 11(100%)  250(96.9%) | 0(0.00%)  8(3.1%) | 0.713 |
| 2021 | Cfa/Csa/Cwb  Cwa | 11(100%)  255(98.5%) | 0(0.00%)  4(1.5%) | 0.846 |

**Table C: Malaria burden comparison between subtypes of tropical regions**

| **Year** | **Climate**  **Sub**  **Classes** | **Tropical Districts with API** | | **p-value** |
| --- | --- | --- | --- | --- |
|  |  | **<1** | **≥1** |  |
| 2015 | Am  Aw | 34 (75.56%)  143 (66.82%) | 11 (24.44%)  71 (33.18%) | 0.167 |
| 2016 | Am  Aw | 36 (80.00%)  156 (72.9%) | 9 (20.00%)  58 (27.1%) | 0.356 |
| 2017 | Am  Aw | 36 (80.00%)  167 (78.04%) | 9 (20.00%)  47 (21.96%) | 0.474 |
| 2018 | Am  Aw | 38 (84.44%)  189 (88.32%) | 7 (15.56%)  25 (11.68%) | 0.309 |
| 2019 | Am  Aw | 38 (84.44%)  194 (90.65%) | 7 (15.56%)  20 (9.35%) | 0.164 |
| 2020 | Am  Aw | 41 (91.11%)  195 (91.12%) | 4 (8.89%)  19 (8.88%) | 0.593 |
| 2021 | Am  Aw | 41 (91.11%)  197 (92.1%) | 4 (8.89%)  17 (7.9%) | 0.513 |
