# Supplementary material for: Climate zones are a key component of the heterogeneous presentation of malaria and should be added as a malariometric for the planning of malaria elimination
Source: PLOS Glob Public Health. 2023 Jun 28;3(6):e0001878. doi: 10.1371/journal.pgph.0001878 (PMC10306175; doi:10.1371/journal.pgph.0001878)
Supplement: Attachment — Submitted filename: Reviewers comments reply 2.docx [file pgph.0001878.s002.docx]

1. We note your Data Availability statement as follows:

"District-wise annual data on malaria incidence (API) from 2016-2021 was obtained from the National Centre for Vector-borne Disease Control (NCVBDC) and is available on request.
Data for the 1km resolution maps of Koppen-Geiger climate classification for the present (1980-2016) as well as future (2100) is available in the public domain from the website (<http://www.gloh2o.org/koppen/>) and is referenced in the study (doi:10.1038/sdata.2018.214)"

We note that you have indicated that a portion of your minimal data from this study are available upon request. PLOS only allows data to be available upon request if there are legal or ethical restrictions on sharing data publicly (<http://journals.plos.org/globalpublichealth/s/data-availability#loc-unacceptable-data-access-restrictions>).

In line with our goal of ensuring long-term data availability to all interested researchers, PLOS’ Data Policy also states that authors cannot be the sole named individuals responsible for ensuring data access (<http://journals.plos.org/globalpublichealth/s/data-availability#loc-acceptable-data-sharing-methods>).

Before we proceed with your manuscript, please address the following relevant prompt in an update Data Availability statement:

**a) If there are ethical or legal restrictions on sharing a de-identified data set, please explain them in detail (e.g., data contain potentially identifying or sensitive patient information) and who has imposed them (e.g., a Research Ethics Committee or Institutional Review Board, etc.). Please provide non-author contact information (phone/email/hyperlink) for a data access committee, ethics committee, or other institutional body to which data requests may be sent. If applicable, please also provide any necessary information which interested researchers would need when requesting access to data in order to obtain the minimal data set for your study.**

**Response:** There are no ethical and legal restrictions on sharing/obtaining these data as neither these have any individual identity (they are pooled data on the district level without patients’ identifiers) nor do they contain any sensitive information related to patients and others. All the data before 2019 are available in the public domain, and data after 2019 are not yet available in the public domain but can be taken on request. Data requests can be made through the website (www.nvbdcp.gov.in/ E-MAIL: [nvbdcpmohfw@nic.in](mailto:nvbdcpmohfw@nic.in).)

Similar questions were asked by the journal office at the time of our earlier publications (given below) and we replied as above.

1: Yadav CP, Gupta S, Bharti PK, Rahi M, Faizi N, Sharma A. India may need an additional metric to assess the endemicity of malaria in low surveillance districts. PLOS Glob Public Health. 2022 Nov 10;2(11):e0000326. doi: 10.1371/journal.pgph.0000326. PMID: 36962502; PMCID: PMC10021988.

2: Yadav CP, Hussain SSA, Gupta S, Bharti PK, Rahi M, Sharma A. Tracking district-level performance in the context of achieving zero indigenous case status by 2027. PLOS Glob Public Health. 2023 Jan 6;3(1):e0001292. doi:10.1371/journal.pgph.0001292. PMID: 36962890; PMCID: PMC10021673.

b) If your minimal data were obtained from a third party (i.e., data not owned or collected by the authors), please explain how others can access or request the specific datasets related to your research. Confirm that others would be able to access or request these data in the same manner as the authors. Please also confirm that the authors did not have any special access or request privileges that others would not have.
We will update your Data Availability statement on your behalf to reflect the information you provide.


Response: Malaria data were taken from National Centre for Vector Borne Diseases Control (NCVBDC), Govt of India. The rest of the data were downloaded from sources in the public domain. As discussed above, malaria data up to 2019 are available in the public domain ([nvbdcpmohfw@nic.in](mailto:nvbdcpmohfw@nic.in)) and data after 2019 may be accessed on request. The request can be made through their website (www.nvbdcp.gov.in/ E-MAIL: [nvbdcpmohfw@nic.in](mailto:nvbdcpmohfw@nic.in).).

Yes, we agree, we both (NIMR and NCVBDC) are government organizations and work for malaria research and control. However, this fact brings no privileges. Other authors may also request data as we did.

2. Please ensure that the Title in your manuscript and the Title in your online submission form are the same.
Response: done

3. Please provide separate figure files in .tif or .eps format only and ensure that all files are under our size limit of 10MB. You may leave the figure captions or legends in the manuscript.

For more information about how to convert your figure files please see our guidelines: <https://journals.plos.org/globalpublichealth/s/figures>

Response: done

4. We have noticed that you have uploaded Supporting Information files, but you have not included a list of legends. Please add a full list of legends for your Supporting Information files after the references list.
Response: All legends have been added now.

5. Some material included in your submission may be copyrighted. According to PLOS’s copyright policy, authors who use figures or other material (e.g., graphics, clipart, maps) from another author or copyright holder must demonstrate or obtain permission to publish this material under the Creative Commons Attribution 4.0 International (CC BY 4.0) License used by PLOS journals. Please closely review the details of PLOS’s copyright requirements here: PLOS Licenses and Copyright. If you need to request permissions from a copyright holder, you may use PLOS's Copyright Content Permission form.

Please respond directly to this email or email the journal office and provide any known details concerning your material's license terms and permissions required for reuse, even if you have not yet obtained copyright permissions or are unsure of your material's copyright compatibility.

Potential Copyright Issues:

Figures 1, 2 and 3: please (a) provide a direct link to the base layer of the map (i.e., the country or region border shape) and ensure this is also included in the figure legend; and (b) provide a link to the terms of use / license information for the base layer image or shapefile. We cannot publish proprietary or copyrighted maps (e.g. Google Maps, Mapquest) and the terms of use for your map base layer must be compatible with our CC-BY 4.0 license.

Note: if you created the map in a software program like R or ArcGIS, please locate and indicate the source of the basemap shapefile onto which data has been plotted.

If your map was obtained from a copyrighted source please amend the figure so that the base map used is from an openly available source. Alternatively, please provide explicit written permission from the copyright holder granting you the right to publish the material under our CC-BY 4.0 license.

Please note that the following CC BY licenses are compatible with PLOS license: CC BY 4.0, CC BY 2.0 and CC BY 3.0, meanwhile such licenses as CC BY-ND 3.0 and others are not compatible due to additional restrictions.

**Response:** Here, we would like to bring to your consideration that the official shape files (**base layer**) for India were procured/purchased by the Institute from the **Survey of India, Ministry of Science & Technology**, **Government of India**, under a collaboration between NIMR, India and Oxford University, UK. The shapefiles were procured for both state boundaries and district boundaries. **The receipt for the purchase has been enclosed as supplementary material.** The API, and koppen classification data were superimposed on the base shape files to create figures 1 to 3 using Arc GIS software.

Regarding the copyright ownership for publishing the figures under the CC BY 4.0 license, we would like to bring to your notice that **Survey of India does not grant individual permissions**. The Survey of India, Ministry of Science and Technology, Government of India, has issued a notification with guidelines (available at [https://onlinemaps.surveyofindia.gov.in/ GeospatialGuidelines.aspx](https://onlinemaps.surveyofindia.gov.in/%20GeospatialGuidelines.aspx)) that **clearly states that there shall be no requirement for prior approval, permission or license to publish these shape-files once purchased**. Only self-certification may be used for adherence to the guidelines.

Similar queries were raised by the journal office at the time of our earlier publications (given below) that were also replied to similarly.

1: Yadav CP, Gupta S, Bharti PK, Rahi M, Faizi N, Sharma A. India may need an additional metric to assess the endemicity of malaria in low surveillance districts. PLOS Glob Public Health. 2022 Nov 10;2(11):e0000326. doi: 10.1371/journal.pgph.0000326. PMID: 36962502; PMCID: PMC10021988.

2: Yadav CP, Hussain SSA, Gupta S, Bharti PK, Rahi M, Sharma A. Tracking district-level performance in the context of achieving zero indigenous case status by 2027. PLOS Glob Public Health. 2023 Jan 6;3(1):e0001292. doi:10.1371/journal.pgph.0001292. PMID: 36962890; PMCID: PMC10021673
